# Characteristics and Outcomes of Mechanically Ventilated COVID-19 Patients—An Observational Cohort Study

**DOI:** 10.1177/0885066620954806

**Published:** 2020-09-02

**Authors:** Martin Krause, David J. Douin, Kevin K. Kim, Ana Fernandez-Bustamante, Karsten Bartels

**Affiliations:** 1 Department of Anesthesiology, 12225 University of Colorado, School of Medicine, Aurora, CO, USA; 2 Department of Medicine, 12225 University of Colorado, School of Medicine, Aurora, CO, USA

**Keywords:** COVID-19, SARS-CoV-2, acute respiratory distress syndrome, mechanical ventilation

## Abstract

**Background:**

The United States currently has more confirmed cases of COVID-19 than any other country in the world. Given the variability in COVID-19 testing and prevention capability, identifying factors associated with mortality in patients requiring mechanical ventilation is critical. This study aimed to identify which demographics, comorbidities, markers of disease progression, and interventions are associated with 30-day mortality in COVID-19 patients requiring mechanical ventilation.

**Methods:**

Adult patients with a confirmed diagnosis of COVID-19 admitted to one of the health system’s intensive care units and requiring mechanical ventilation between March 9, 2020 and April 1, 2020, were included in this observational cohort study. We used Chi-Square and Mann-Whitney U tests to compare patient characteristics between deceased and living patients and multiple logistic regression to assess the association between independent variables and the likelihood of 30-day mortality.

**Results:**

We included 85 patients, of which 20 died (23.5%) within 30 days of the first hospital admission. In the univariate analysis, deceased patients were more likely ≥60 years of age (p < 0.001), non-Hispanic (p = 0.026), and diagnosed with a solid malignant tumor (p = 0.003). Insurance status also differed between survivors and non-survivors (p = 0.019). Age ≥60 and malignancy had a 9.5-fold (95% confidence interval 1.4-62.3, p = 0.020) and 5.8-fold higher odds ratio (95% confidence interval 1.2-28.4, p = 0.032) for 30-day mortality after adjusted analysis using multivariable logistic regression, while other independent variables were no longer significant.

**Conclusions:**

In our observational cohort study of 85 mechanically ventilated COVID-19 patients, age, and a diagnosis of a solid malignant tumor were associated with 30-day mortality. Our findings validate concerns for the survival of elderly and cancer patients in the face of the COVID-19 pandemic in the United States, where testing capabilities and preventative measures have been inconsistent. Preventative efforts geared to patients at risk for intensive care unit mortality from COVID-19 should be explored.

## Background

The global pandemic of novel coronavirus disease 2019 (COVID-19) caused by severe acute respiratory syndrome coronavirus 2 (SARS-CoV-2) began in Wuhan, China, in December 2019, and has since spread worldwide.^1^ Patients with COVID-19 have high rates of hospitalization and intensive care unit (ICU) admission.^2^ As of July 11th, 2020, there have been nearly 13 million confirmed cases and more than 500,000 deaths worldwide.^3^ Notably, the United States has more than 3 million confirmed cases, which is more than any other country and currently exceeds the second-ranked country Brazil by more than 1 million.^3^

Similar to other coronavirus infections, patients infected with SARS-CoV-2 have a variable clinical course.^4,5^ While many patients do not require hospitalization, nearly 8 in 10 hospitalized patients require supplemental oxygen and as many as one-third of these patients require some form of mechanical ventilation.^5–7^ Virtually all COVID-19 patients who require mechanical ventilation meet the criteria for acute respiratory distress syndrome (ARDS).^8,9^ ARDS is a progressive, life-threatening inflammatory pulmonary process characterized by diffuse alveolar damage and rapid clinical deterioration.^8,10^ However, clinicians worldwide have noted that COVID-19 patients have a different disease trajectory than most other ARDS patients.^2,4,11^

Many patient characteristics are known to be associated with an increased risk of a severe disease course. These include hypertension,^2,5,12^ diabetes mellitus,^12,13^ and obesity.^14,15^ Given the variable ability to prevent, test for, and treat COVID-19 in the United States,^16^ understanding of factors associated with mortality in patients requiring scarce critical care and mechanical ventilation resources is highly relevant. We sought to validate findings from other countries by analyzing a COVID-19 patient cohort from a large United States healthcare system. Using an observational cohort study approach, we describe demographics, comorbidities, data on disease progression, interventions, and mortality of patients with a laboratory-confirmed COVID-19 diagnosis that required admission to the ICU and mechanical ventilation. Our hypothesis was that certain demographics, patient characteristics, and differences in disease progression and management would be associated with 30-day mortality.

## Methods

Institutional Review Board approval (Colorado Multiple Institutional Review Board #20-0677) was obtained, and the requirement for informed consent was waived. Data were collected retrospectively for any events prior to this date and prospectively going forward. This manuscript adheres to the Strengthening the Reporting of Observational Studies in Epidemiology (STROBE) guideline.^17^

### Aim, Design, and Setting

The aim of this study was to provide early information on demographics, chronic comorbid conditions, disease progression, and treatment interventions associated with 30-day mortality in a cohort of COVID-19 patients requiring mechanical ventilation in a large United States health care system comprised of 12 hospitals. This study was designed as an observational cohort study using data collected from the electronic health record (EHR) and manual chart review as needed.

### Participants, Covariates, and Outcomes

**Figure 1.**
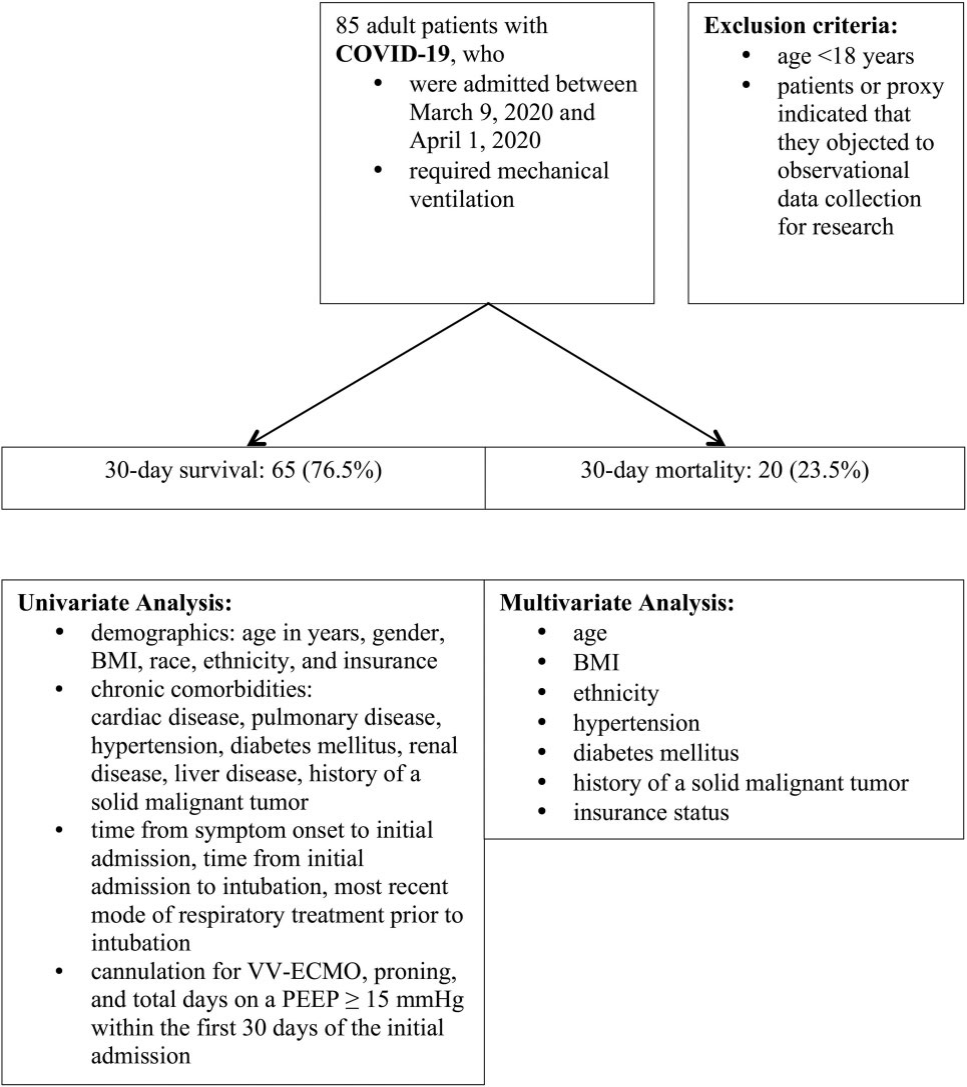
Study flowchart. BMI: body mass index, VV-ECMO: veno-venous extracorporeal membrane oxygenation, PEEP: positive end-expiratory pressure.

All adult patients diagnosed with COVID-19 and admitted to one of the health system’s ICUs, who required mechanical ventilation between March 9, 2020 and April 1, 2020, were eligible for inclusion. Patients were excluded if they were <18 years old and if they or their proxies indicated that they objected to observational data collection for research (Figure 1).

Applicable patient demographics were obtained from the medical record. Demographics included: age in years, self-reported gender, body mass index (BMI) in kg/m^2^, race, ethnicity, and insurance status. Chronic comorbidities were selected based on previously reported findings using pre-existing International Classification of Diseases codes (ICD-9 or ICD-10). These included: cardiac disease, pulmonary disease, hypertension, diabetes mellitus, renal disease, liver disease, and a history of a solid malignant tumor. Important information about disease progression and interventions were collected by manual chart review: time from symptom onset to initial admission, time from initial admission to intubation, the most recent mode of respiratory treatment prior to intubation (nasal cannula, face mask, heated high flow nasal cannula, or non-invasive ventilation), as well as cannulation for veno-venous extracorporeal membrane oxygenation (VV-ECMO), proning, and total days with a positive end-expiratory pressure (PEEP) ≥ 15 mmHg^18^ within the first 30 days of the initial admission for COVID-19. Thirty-day mortality following the first admission for COVID-19 was assessed based on the date of death as recorded in the EHR.

### Statistical Analysis

We summarized data by means of descriptive statistics. Continuous data were evaluated for normality of distribution using the Kolmogorov-Smirnov test. Univariate comparisons were made using Pearson’s Chi-Square and Mann-Whitney U tests. Next, we checked for an association between patient demographics, comorbidities, information on disease progression, or interventions, and the likelihood of 30-day mortality by fitting a multiple logistic regression model. Based on published findings and the significant results from our univariate results,^12,14,15,19–21^ we included age, BMI, ethnicity, hypertension, diabetes mellitus, insurance status, and a history of a solid malignant tumor as covariates in our model. The software program SPSS, Version 26 (IBM Corporation, Armonk, NY) was used for statistical analysis.

## Results

We included 85 patients on mechanical ventilation, who had tested positive for COVID-19 in this study. Of these patients, 20 died within 30 days of their index admission (23.5%). Baseline demographics, patient comorbidities, data about disease progression, and treatment interventions are summarized in Table 1. Deceased patients were more likely ≥60 years of age vs. younger (40.9% vs. 4.9% of these had died, p < 0.001), diagnosed with a solid malignant tumor vs. not (63.6% vs. 17.6% of these had died, p = 0.003), and non-Hispanic vs. Hispanic (31.0% vs. 7.4% of these had died p = 0.026). Insurance status also differed between survivors and non-survivors: 30-day mortality was 12.5% for Managed Care, 40.6% for Medicare, and 14.3% for Medicaid and others (p = 0.019). There were no other significant differences in demographics, comorbidities, disease progression, and treatment interventions.

**Table 1.** Patient Demographics, Comorbidities, Data on Progression of Disease, and Interventions.

Characteristic	All	Alive	30-day mortality	p-value
Total—no.	85	65 (76.5)	20 (23.5)	
Age—no. (%)				<0.001
≥ 60 years	44	26 (59.1)	18 (40.9)	
<60 years	41	39 (95.1)	2 (4.9)	
Gender—no. (%)				0.276
Men	57	46 (80.7)	11 (19.3)	
Women	28	19 (67.9)	9 (32.1)	
Body mass index—no. (%)				0.809
<30 kg/m^2^	30	22 (73.3)	8 (26.7)	
≥30 to <35 kg/m^2^	24	18 (75.0)	6 (25.0)	
≥35 kg/m^2^	31	25 (80.6)	6 (19.4)	
Race—no. (%)				0.896
Caucasian or White	42	32 (76.2)	10 (23.8)	
African American or Black	22	16 (72.7)	6 (27.3)	
Multiple races or other	21	17 (81.0)	4 (19.0)	
Ethnicity—no. (%)				0.026
Hispanic	27	25 (92.6)	2 (7.4)	
Not Hispanic	58	40 (69.0)	18 (31.0)	
Insurance status—no. (%)				0.019
Managed care	32	28 (87.5)	4 (12.5)	
Medicare	32	19 (59.4)	13 (40.6)	
Medicaid, self pay, indigent, or other	21	18 (85.7)	3 (14.3)	
Chronic cardiac disease—no. (%)				0.180
Yes	30	20 (66.7)	10 (33.3)	
No	55	45 (81.8)	10 (18.2)	
Hypertension—no. (%)				0.274
Yes	58	42 (72.4)	16 (27.6)	
No	27	23 (85.2)	4 (14.8)	
Chronic pulmonary disease—no. (%)				0.180
Yes	30	20 (66.7)	10 (33.3)	
No	55	45 (81.8)	10 (18.2)	
Diabetes mellitus—no. (%)				1.000
Yes	33	25 (75.8)	8 (24.2)	
No	52	40 (76.9)	12 (23.1)	
Preexisting renal disease—no. (%)				1.000
Yes	24	18 (75.0)	6 (25.0)	
No	61	47 (77.0)	14 (23.0)	
Chronic liver disease—no. (%)				0.504
Yes	15	10 (66.7)	5 (33.3)	
No	70	55 (78.6)	15 (21.4)	
History of solid malignant tumor—no. (%)				0.003
Yes	11	4 (36.4)	7 (63.6)	
No	74	61 (82.4)	13 (17.6)	
Days from symptom onset to admission				0.100
– median (25th percentile; 75th percentile)	6 (4; 10)	7 (5; 10)	5 (2; 9)	
Hours from admission to intubation – median (25th percentile; 75th percentile)	33 (5; 76)	33 (5; 75)	29 (9; 99)	0.988
Respiratory therapy prior to intubation—no. (%)				0.731
Nasal cannula	14	12 (85.7)	2 (14.3)	
Face mask	51	38 (74.5)	13 (25.5)	
Heated high flow nasal cannula	16	12 (75.0)	4 (25.0)	
Non-invasive ventilation	2	1 (50.0)	1 (50.0)	
Unknown	2	2 (100)	0 (0)	
Days on PEEP ≥ 15 mmHg within 30 days of admission – median (25th percentile; 75th percentile)	1 (0; 4)	0 (0; 4)	2 (0; 6)	0.154
VV-ECMO within 30 days of admission—no. (%)				0.579
Yes	3	3 (100)	0 (0)	
No	82	62 (75.6)	20 (24.4)	
Proned within 30 days of admission—no. (%)				0.795
Yes	51	40 (78.4)	11 (21.6)	
No	34	25 (73.5)	9 (26.5)	

The p-values signify exact 2-sided Chi-Square test results for binary outcomes and Mann-Whitney U test results for continuous outcomes. PEEP—positive end-expiratory pressure, VV-ECMO—veno-venous extracorporeal membrane oxygenation.

Adjusted results from a multivariable regression model are summarized in Table 2. After adjusting for age, hypertension, insurance status, ethnicity, BMI, diabetes mellitus, and any history of solid malignant tumor, age ≥60 had 9.5-fold higher odds ratio (95% confidence interval 1.4-62.3, p = 0.020) and malignancy had 5.8-fold higher odds ratio (95% confidence interval 1.2-28.4, p = 0.032) for 30-day mortality.

**Table 2.** Multiple Logistic Regression Analysis: Odds Ratio and 95% Confidence Interval for 30-Day Mortality.

Characteristic	Odds ratio	95% confidence interval	p-value
Age			
≥ 60 years vs <60 years	9.451	1.433-62.316	0.020
Ethnicity			
Hispanic vs non-Hispanic	0.281	0.044 -1.802	0.181
Insurance status			
Managed Care			0.745
Medicare	1.098	0.218-5.529	0.910
Medicaid	2.064	0.294-14.481	0.466
Body mass index			
<30 kg/m^2^			0.855
≥ 30-35 kg/m^2^	1.124	0.229-5.519	0.886
≥35 kg/m^2^	1.541	0.332-7.155	0.581
Hypertension	1.979	0.450-8.703	0.366
Diabetes mellitus	0.748	0.195-2.867	0.671
History of solid malignant tumor	5.756	1.165-28.447	0.032

For insurance status the reference group is “managed care”.

## Discussion

Age, malignancy, insurance status, and ethnicity were associated with increased 30-day mortality in mechanically ventilated COVID-19 patients upon univariate analysis. After fitting a multiple logistic regression model, age ≥60 years and malignancy remained associated with 30-day mortality in this United States-based patient cohort. Given that to date, the United States has more confirmed COVID-19 cases than any other country in the world,^3^ and that the capability to prevent and treat COVID-19 has been variable,^16^ our results contribute to the emerging knowledge about the critical care implications of the COVID-19 pandemic. Furthermore, these data confirm our hypothesis that certain patient characteristics would also be associated with increased mortality in our patient sample obtained from a large United States health system.

Similar to our results, initial reports from China and Italy recognized age as a risk factor for increased mortality.^2,5^ Improved protective measures in elderly patients may decrease overall-mortality of COVID-19-infected patients.^22^ However, which specific protective measures should be recommended for at-risk populations such as the elderly, and the determination and degree to which such interventions lead to reductions of COVID-19 related mortality requires additional research.^23^

In addition to older age, patients diagnosed with solid malignant tumors displayed increased mortality. In line with this finding, a retrospective study from Wuhan, China, concluded that cancer patients demonstrate poorer outcomes after COVID-19 infections.^20^ Prevention and control measures for patients with cancer are, therefore, warranted.^24^

Our 30-day mortality in a tertiary care center in ventilated patients (23.5%) is lower than previously reported mortality in ventilated patients in Wuhan, China (65.7%), and critically ill patients in Washington State (67%).^5,13^ We speculate that this finding could, in part, reflect a positive correlation between mortality and a heavier healthcare burden in proximity to epicenters of this pandemic,^25^ but could also be due to factors such as different follow-up times and modes of data collection.

### Limitations

This study has several limitations. We performed an observational study, and unaccounted confounders may be present.^26^ However, our study included patients admitted from 12 different hospitals in our health system, which hopefully ensured the diversity of the population studied. Furthermore, results from our Colorado-based study may not be representative of other regions in the United States. Inferences should, therefore, be made with caution. Finally, our study assessed 30-day mortality in only 85 ventilated patients. A larger number of subjects could potentially identify other characteristics or laboratory findings associated with mortality.

## Conclusion

In this observational cohort study of 85 COVID-19 positive patients, who required mechanical ventilation, we report an association between age ≥60 years and a history of solid malignancy with 30-day mortality. Improved protective measures in patients of older age and a history of cancer may decrease overall-mortality of COVID-19, but further randomized controlled trials are warranted to confirm this association.
